# The International Human Xenotransplantation Inventory: Current Data and Future Directions

**DOI:** 10.1097/TP.0000000000005367

**Published:** 2025-04-08

**Authors:** Xiaowei Hu, Wayne J. Hawthorne, Leo Buhler

**Affiliations:** 1 Division of Anaesthesiology, Department of Acute Care Medicine, Geneva University Hospitals, Geneva, Switzerland.; 2 Faculty of Science and Medicine, University of Fribourg, Fribourg, Switzerland.; 3 Department of Surgery, Westmead Hospital, School of Medical Sciences, University of Sydney, Westmead, NSW, Australia.

## Abstract

The global demand for organ transplantation outpaces supply, necessitating innovative solutions. Xenotransplantation, using animal organs, cells, and tissues, is a promising solution to address the organ shortage. The World Health Organization and the International Xenotransplantation Association collaboratively established an online inventory in 2006 (www.humanxenotransplant.org) to catalog human xenotransplantation practices. The inventory, managed successively by the Geneva University Hospital and the Sichuan Provincial People’s Hospital, aligns with the World Health Organization directives for transparency and best practices in the field of transplantation. Relevant data have been regularly collected from numerous sources (scientific publications, congresses, press articles, and declarations of International Xenotransplantation Association members) by a dedicated team in Switzerland and China, ensuring rigorous verification. The initial information is used to create a first entry in the database, which is then completed when more details become available. As of May 2024, the inventory contained 54 entries of distinct xenotransplantation procedures undertaken on humans. From these data, various trends can be observed over the past 2 decades regarding the type of transplantation, their regulation status, and the source animal. Notably, recent high-profile cases of solid organ transplantation involving kidneys and hearts were made feasible through years of progressive xenotransplantation research and ongoing changes to regulations. Recent clinical applications of solid organ xenotransplantation suggest that more clinical procedures may soon follow for patients with end-stage kidney or heart disease or diabetes. Future perspectives advocate for increased funding and expansion of the current registry or its potential integration into a larger and more broadly internationally recognized registry, such as the Global Observatory on Donation and Transplantation.

## THE NEED FOR A XENOTRANSPLANTATION INVENTORY

The global demand for organ transplantation continues to rise, outpacing the supply despite an increasing number of transplant procedures being performed. This shortage remains a critical barrier to effectively treating many individuals with end-stage organ failure. In Europe alone, an average of 20 patients die each day while awaiting a transplant.^1^ This imbalance between the availability of donors and the growing need for organs is further compounded by the escalating prevalence of chronic diseases, which not only increases the demand for organs but also diminishes the pool of potential donors.^2^

To address this pressing organ shortage crisis, xenotransplantation provides a long-term promising solution. This solution aims to use organs from animals and holds the potential to provide a consistent, safe, and efficacious supply of organs, offering a viable solution to this crisis. By having a more abundant source of organs, it becomes feasible to conduct transplantation at earlier stages of end-organ failure, which will significantly enhance the quality of life and overall outcomes for patient.^3^ However, the clinical application of xenotransplantation encounters significant challenges, primarily stemming from pronounced immunological and hematological disparities between species. Additionally, the perceived potential risk of xenozoonosis, the transmission of infectious animal diseases to humans, remains a concern, especially given the current need for immunosuppressive regimens to prevent graft rejection.

To establish guidelines for best practices in the expanding domain of xenotransplant research, the World Health Organization (WHO) made several resolutions regarding its practice. At the outset of current xenotransplant research, several recommendations were made regarding xenotransplantation in the resolution WHA57.18 on human organ and tissue transplantation in 2004^4^ and resulted in the gathering of a panel of experts in April 2005 in Geneva, Switzerland. During this advisory consultation, the need for an international inventory to catalog human xenotransplantation practices was proposed. This along with the encouragement of Members States to establish appropriate regulatory systems regarding clinical practices, weigh their risks versus benefits, set standards for animal sourcing and procedures, ensure robust surveillance, promote transparency, and raise public awareness.^5^ This framework was established with the main aim being to ensure the safe and effective implementation of clinical xenotransplantation practice while mitigating any potential public health risks.

In collaboration with the International Xenotransplantation Association (IXA) and the WHO, an online inventory (www.humanxenotransplant.org) was established in 2006 as proposed and supported by the WHO and The Transplantation Society (TTS). The inventory was established and managed by the Geneva University Hospital until its redesign in 2020. At its creation, this repository meticulously registered all documented xenotransplantation procedures conducted on humans since 1995. In 2020, the website was redesigned to provide more online information and be more in line with current website publishing, and its management and hosting were subsequently transferred to the Sichuan Provincial People’s Hospital in Chengdu, China.

The inventory aligned with the directives of WHO as outlined in various resolutions, including the 2005 advisory consultation and the Changsha Communiqués of 2008 and 2018,^6,7^ aiming to enhance accessibility and transparency in documenting xenotransplantation practices. Its main goal was to make information more accessible to the broader transplantation community, the public, and officials, fostering transparency, thereby promoting best practices within the xenotransplantation field. Moreover, the inventory seeks to address the critical shortage of available human organs by facilitating a structured platform that catalogs and updates xenotransplantation procedures, ultimately contributing to advancements in this field. The recent progress, particularly in using genetically modified pigs for xenotransplantation, underscores the significance of maintaining an updated and comprehensive inventory to reflect evolving practices and research breakthroughs.

The content of the inventory has been described in numerous scientific articles. The inaugural article published in 2010 unveiled the inventory and comprehensively presented all recorded clinical practices of human xenotransplantation procedures between 1995 and 2010.^8^ A more recent update, published in 2021, detailed applications performed from 2010 to 2020.^9^ More importantly, starting in late 2021, a series of groundbreaking achievements thrust xenotransplantation into the limelight after the pioneering transplantations of a 10-gene-edited porcine heart into a live patient and several transgenic pig kidneys and 2 hearts into brain-dead patients. A third article was then published in 2023 to account for this significant recent progress.^10^

## DATA COLLECTION FOR THE INVENTORY

The updated inventory contains information gathered from various sources, including scientific publications, press releases, presentations at academic conferences, news articles, and declarations from IXA members. Additionally, an electronic form is accessible online for submitting new data or advising of new cases that have not yet been identified.

A comprehensive online search is regularly conducted to screen for new procedures, using specific keywords on platforms such as Google for news articles, PubMed for scientific publications, and clinicaltrials.gov for new clinical trials. As the current xenotransplantation community remains relatively small and close-knit, a substantial amount of information is also directly shared between IXA members.

The team in charge of this process includes members located at the Geneva University Hospital, Geneva, Switzerland (X.H.), the Fribourg Cantonal Hospital and University of Fribourg, Fribourg, Switzerland (L.B.), and the Sichuan Provincial People’s Hospital, Chengdu, China (Mrs Z. Geng).

Once a new clinical application of xenotransplantation is identified, research is conducted by the team to gather and verify relevant information available at the time of initial identification, followed by updating once more information becomes available. For instance, recent practices over the past few years have involved high-profile cases of solid organ transplants, with an initial source of data being the institution-issued press releases or exclusive press articles. Relevant information available from these initial sources was then collected and an initial entry was created in the database using these data. The remaining details were later completed when the associated scientific article was published, usually a few months later.

The database includes a large amount of information regarding the details of the practice, with various questions regarding the institutions involved, the clinical trial, animal source, biological safety testing and monitoring, patient follow-up, and official oversight and approval. The complete list of questions can be found on the website. It is worth noting that some secondary questions from the questionnaire may not be completed, as some information is sometimes not officially divulged, even in academic articles.

## KEY TRENDS IN HUMAN XENOTRANSPLANTATION (1990–2024)

As of May 2024, the inventory contained 54 documented entries of distinct xenotransplantation practices undertaken on humans, performed between 1990 and 2024 (Table 1).

**TABLE 1.** Numbers and proportions of type of transplantation, main source of information, regulation status, and source animal for current entries into the registry

	N	%
Type of transplantation		
Cells	27	50%
Tissue	5	9.3%
Organ	14	25.9%
Extracorporeal transfusion	8	14.8%
Main source of information		
Scientific publication	27	50%
Congress presentation	4	7.4%
Internet	10	18.5%
Press release	12	22.2%
Other	1	1.9%
Regulation status		
With national regulation	31	57.4%
Without national regulation	23	42.6%
Source animal (a few cases involved >1 species, causing the total number to exceed 100%)		
Pig	44	81.5%
Other mammal	10	18.5%
Non-mammal	2	3.7%
Unknown	2	3.7%
Total	54	100%

**FIGURE 1.**
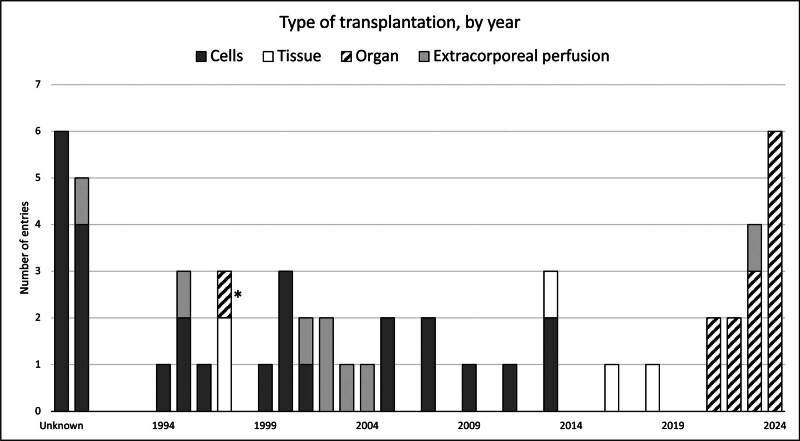
Type of transplantation, by year, from 1990 to 2024. The asterisk outlines a case of heart xenotransplantation unofficially conducted in India (see the text).

Figure 1 displays the number of distinct xenotransplantation procedures conducted on humans per year (or starting year if the trial is extended across multiple years), spanning from 1990 to 2024. These numbers represent independent sets of xenotransplantation trials and do not reflect the absolute number of patients involved in any one study. A few discernible trends emerge from this graph. Notably, there was a remarkable surge in extracorporeal perfusion procedures, primarily encompassing hepatocytes for acute liver failure treatment, performed from 2000 to 2004. Cellular transplantation maintained a consistent presence from the first records until 2013, predominantly featuring islets of Langerhans trials for the treatment of type 1 diabetes. These occurred alongside various other cell types for diverse indications, such as treatments of Parkinson’s and Huntington’s disease using fetal porcine neuronal cells.

An intriguing observation is the clustering of cases allocated as unknown (displayed on the far-left side of Figure 1); they are categorized as such due to a lack of documentation—lacking support from peer-reviewed scientific publications, often of unknown timing—and exclusively pertain to cellular transplantation, occasionally associated with uncertain indications (eg, xenotransplantation of sheep cells from reproductive glands for antiaging purposes). The tendency to perform cellular transplants may well be attributable to the relatively simpler nature of cellular transplantation when compared with the logistical, technical, and financial complexities inherent in solid organ transplants. Disturbingly, the majority of these procedures were performed very early on in the timeline (in the 1990s) and had little, if any, regulatory approval or oversight being predominantly performed in less regulated jurisdictions.

Moreover, the latter year segment of the chart illustrates the upsurge in recent solid organ transplantation cases, particularly involving kidneys and hearts, made feasible through years of progressive xenotransplantation research specifically from preclinical testing in nonhuman primate models. The most recent case of an auxiliary liver xenotransplant in a 71-y-old patient, performed in May 2024 in China, is also an astonishing addition to current progress. With many decades of focused xenotransplantation research and underpinning from the constant engagement of various jurisdictions regulatory bodies and overarching efforts from the IXA in conjunction with the WHO to develop appropriate guidance. However, an exception stands out from the rest in the form of an infamous heart xenotransplantation case conducted in India in 1997 (marked with an asterisk in Figure 1), resulting in the patient’s demise after 7 d and the subsequent prosecution and conviction of the surgeon involved.^11^

The primary goal of the inventory has been to promote good medical practice, notably through the encouragement of the adoption of appropriate regulation. Additionally, the WHO, along with the IXA, established a number of committees following WHO Resolution 57.18 and arranged the WHO Xenotransplantation Advisory Consultation, which was held in Geneva in April 2005. The role of each Member State was more comprehensively defined during this meeting, with the importance of xenotransplantation being well established as an alternative to help improve the increasing worldwide gap between organ demand and supply. The main points raised by the committee included that “Member States should be responsible for tracking of patients once they were transplanted, undertake an inventory of xenotransplantation practices and that xenotransplantation should only occur if there were effective regulatory systems in place.” Another important directive among a number of additional points was that the WHO should be notified about any public health issues as a result of any clinical xenotransplantation.

The first WHO Global Consultation on Regulatory Requirements for Xenotransplantation Clinical Trials was held in Changsha, China, in 2008. The resulting Changsha Communiqué described 10 guiding principles and 20 recommendations for consideration by the WHO, Member States, Investigators, and proposers of clinical trials using xenotransplantation products. This provided significant guidance and solidified the links between the IXA, TTS, and the WHO, and enforced the need for the registry.

**FIGURE 2.**
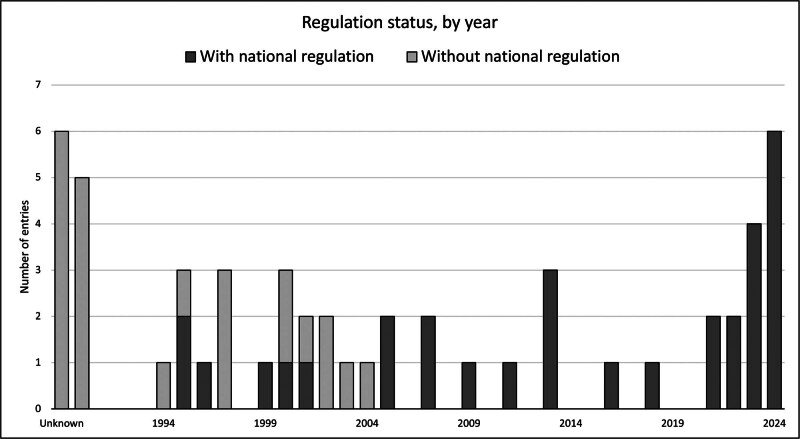
Practices undertaken, by year, from 1990 to 2024, indicate the regulatory status of the jurisdiction when and where the case was undertaken.

Figure 2 outlines the regulatory status of clinical practices recorded. At the time of the Geneva consultation that initiated the inventory’s inception in 2005, the majority of previous cases lacked official assessment or endorsement through official approvals due to a lack of legislation by regulatory authorities in the various jurisdictions. By identifying actors involved in practicing xenotransplantation procedures without official oversight, it was hoped that it would motivate groups, institutions, and private companies to seek official evaluation and approval, thus reducing the risk of negative incidences such as the potential emergence of a xenozoonotic infection. Incidentally, all the cases documented from 2005 onward have coincided with the inventory establishment and the regulatory approval from an official institution. It is worth noting that the few undocumented cases have lacked official approval, and some were performed in jurisdictions that also lacked appropriate regulatory oversight.

**FIGURE 3.**
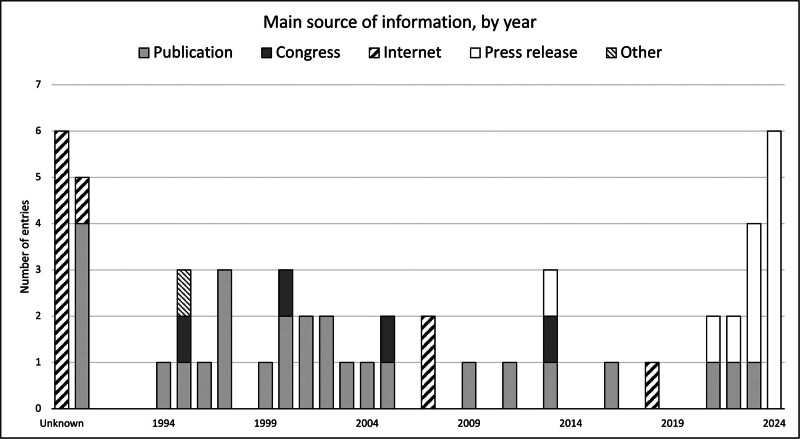
Main source of information, by year, from 1990 to 2024. Publication refers to peer-reviewed scientific articles, and Congress also includes other academic conferences, etc.

As described in the Methods section, relevant information regarding any new practice is regularly collected from diverse sources, as shown in Figure 3. The figure illustrates the main source for every registry entry and highlights that a significant majority of cases are supported by at least 1 peer-reviewed scientific publication. Some instances were initially presented during transplantation congress and may have constituted the foundation for ulterior published practices.

Similarly to previously observed occurrences, practices of unknown timing typically surface through internet webpages, often providing inadequate information regarding the specifics of the practice. However, information sourced through webpages can also be legitimate and appropriately documented, typically when originating from a private pharmaceutical or biotechnical company. A few of the most recent trials are still documented by information gathered from press releases and media articles, as their related peer-reviewed scientific articles are still in preparation or awaiting publication. Finally, 1 case performed in 1995 was self-documented by its principal investigator using the online questionnaire as per IXA recommendations, proving that this is possible and easy enough to be done.

**FIGURE 4.**
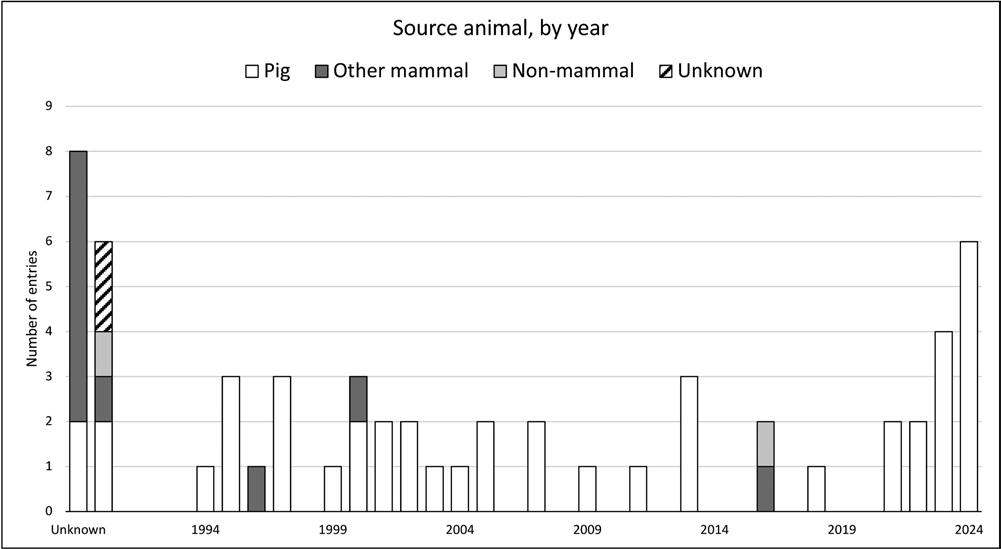
Source animal, by year, from 1990 to 2024. A few cases involved multiple different source animals, hence the increased total number in comparison with other figures.

The choice of animal source used for xenotransplantation to humans has been diverse and has changed over time as seen in Figure 4. Initially, nonhuman primates were chosen due to their close evolutionary proximity to humans, resulting in several trials of organ xenotransplantation in the previous century. Notably, the case of “Baby Fae” in 1983, in which an infant girl with a life-threatening congenital heart disease received a successful baboon heart xenotransplant but sadly died 20 d posttransplant of graft rejection. This particular case drew significant public attention to the scarcity of available organs for infants in need of transplantation.^12^ Gradually, the pig emerged as the most promising candidate for xenotransplantation, owing to the obvious and overwhelming ethical issues along with inherent logistical and technical advantages over nonhuman primates and other animals. This evolution is demonstrated in Figure 4, affirming the preeminence of pigs as the preferred animal in the modern era of clinical xenotransplantation.

The data set also illustrates a resurgence of clinical xenotransplantation practice over the past 3 y, in comparison with a relatively low activity period in the preceding few years. However, significant progress in nonhuman primate preclinical models was achieved during this time, enabling the initiation of current clinical trials by a few groups involved in preclinical kidney and heart transplants, with even more trials expected in the near future. As the number of projected clinical trials is increasing, the urgency for comprehensive collection of all relevant data becomes even more necessary. It is necessary to collate these data to satisfy the growing attention and the need for accurate source data to be able to provide accurate reporting on the field and satisfy the requirements as first set out by the WHO directives for transparency and best practices in the field of transplantation. However, the increasing number of cases could eventually become too burdensome for the current structure of this inventory; without dedicated funding and staffing, it will become untenable.

## THE NEED FOR ITS INTEGRATION INTO A BROADER REGISTRY

The recent upsurge of clinical applications of solid organ xenotransplants in both live and brain-dead patients indicates that increasing numbers of xenotransplants will likely be tested in various centers around the world for patients with end-stage kidney or heart disease and diabetes. Therefore, the current xenotransplant inventory would benefit from being reinvigorated with stronger international support, including financial support for undertaking the significant tasks required for custodianship of this registry or alternatively its potential inclusion into a larger internationally supported and well-prescribed registry.

One such registry and a model to potentially emulate is that of the Global Observatory on Donation and Transplantation. It represents the most comprehensive source to date of worldwide data concerning activities in organ donation and transplantation derived from official sources, as well as information on legal and organizational aspects.

The Global Observatory on Donation and Transplantation is based on the collaboration between the WHO and the Spanish Transplant Organization, Organización Nacional de Trasplantes, and meets the requirements as stipulated by the WHO for transparency and best practices in the field of transplantation. As such, with support from the IXA, TTS, and WHO, such a transition could be undertaken.

In view of future developments and broader applications of clinical xenotransplantation, it seems indicated that the current xenotransplant inventory should be integrated into such an internationally recognized registry. This seems the most logical path unless more funds are provided to expand and reinvigorate the current registry and a significant increase in oversight is provided.
